# The Role of Length of Nerve Grafts in Combination with Free Functional Muscle Transplantation for Brachial Plexus Injury: A Single-Center Experience

**DOI:** 10.3390/jpm14090940

**Published:** 2024-09-04

**Authors:** Michael H. J. Becker, Franz Lassner, Kay W. Nolte, Gary A. Brook, Joachim Weis

**Affiliations:** 1 Pauwelsklinik, Boxgraben 56, 52064 Aachen, Germany; becker@pauwelsklinik.de; 2 Institute of Neuropathology, RWTH Aachen University Hospital, Pauwelsstrasse 30, 52074 Aachen, Germany; knolte@ukaachen.de (K.W.N.); gbrook@ukaachen.de (G.A.B.); jweis@ukaachen.de (J.W.)

**Keywords:** brachial plexus injury, free neurovascular muscle transfer, free functioning muscle transfer, long nerve grafts

## Abstract

Purpose: Extensive lesions of the brachial plexus, or late cases, require free functional muscle grafts because the expected recovery time exceeds the critical threshold of 1.5 years, beyond which irreversible damage may be expected in the distal nerve stump and in the muscle. The reconstructive concept consists of a two-stage procedure where, in the first step, a nerve transfer is performed (from ipsi- or contralateral donor nerves). In the second step, after successful axonal regeneration within the graft has been confirmed, a free muscle transfer is performed. These grafts often exceed 40 cm in length, particularly for contralateral transfers. The purpose of this study was to assess whether robust motor recovery could be supported by such long nerve grafts. Methods: From April 2004 to April 2023, a total of 327 free functional muscle transfers were performed, the nerve graft length ranging from 0 cm (direct coaptation) to 90 cm (serial grafts). Motor recovery was evaluated 1.5 years after surgery according to the MRC scale. Results: A total of 208 patients were available for follow up. Direct coaptation yielded the best results, with 83% of patients reaching an M3 or M4 level of muscle strength. With the application of long (30–60 cm) grafts, 73% of the patients were classified as M3 or M4. The application of serial nerve grafts, however, only resulted in 18% of patients achieving a motor recovery rating of M3. Conclusions: These findings demonstrate that robust motor regeneration is supported by long (30–60 cm) nerve grafts, whereas serial nerve grafting results in a marked reduction in the quality of regeneration.

## 1. Introduction

Proximal injury to peripheral nerves (high lesions), in particular to the brachial plexus, poses a challenge for surgical reconstruction because the expected recovery time often exceeds the critical threshold of 1.5 years (authors’ own unpublished observations). The ability of skeletal muscles to recover after such long periods of denervation deteriorates rapidly following the irreversible degeneration of neuromuscular junctions. Assuming the rate of axonal regeneration to be 1 mm per day [1], and a distance of 80 cm between the Th1 spinal nerve root and the palmar region, a regeneration period of approximately 800 days may be expected, which far exceeds the critical time required for reinnervation of intrinsic hand muscles. For extrinsic hand musculature, the neuromuscular junctions are located in the proximal third of the forearm, resulting in a calculated regeneration distance of approximately 50 cm, equivalent to a 500-day regeneration period. Successful reinnervation of these muscles would therefore be feasible if surgical reconstruction took place shortly after the trauma occurred. This, however, is often not possible because the injury occurs as part of a polytrauma incident and the treatment of concomitant life-threatening injuries takes precedent [2]. For these cases, where target motor end organs may have irreversibly deteriorated over time [2], as well as for all patients who present for surgical reconstruction of nerve injury after a significant delay, the option of a free muscle transfer with neuromuscular connection (i.e., the replacement of irreversibly damaged muscles) is available [3,4,5,6]. For such a strategy, a two-stage procedure is employed with the aim of minimizing the duration of muscle denervation. Following a large lesion (including root avulsions), there may be no local motor nerve available and a contralateral nerve transfer has to be considered. A suitable donor nerve must be selected (see Table 1 for a list of possible donor nerves) which is to undergo coaptation, as part of the first stage of the procedure, with a nerve graft (sural nerve or saphenous nerve), the distal end of which is to be placed in the region of the planned neurovascular anastomosis. At the anticipated time at which the first regenerating axons should have arrived at the distal end of the nerve graft (verified by biopsy), the second stage, consisting of a muscle transfer, can be carried out. The post-operative time between stage I and stage II ranged from 12 to 18 months. The estimated point of time when regenerating axons were expected to be present at the distal end of the graft was based on a regenerating speed of 1 mm per day, plus an extra 4 weeks to account for the anticipated time required for regenerating axons to overcome the coaptation site. To restore grip function of the hand, graft distances of 30–40 cm usually have to be bridged, for which ipsilateral donor nerves may be used. In the case of injuries with root avulsions, it is recommended that donor nerves should also be obtained from the opposite side (i.e., contralateral transfers). In a few cases, such surgery requires the use of serial grafts. 

In view of the fact that a certain loss of axons takes place at each coaptation site [7,8], even poorer regeneration results may be expected with serial nerve grafts. In the present study, we have investigated whether the length of the (non-serial) graft also has an influence on the quality of regeneration.

## 2. Material and Methods

**Figure 1:**
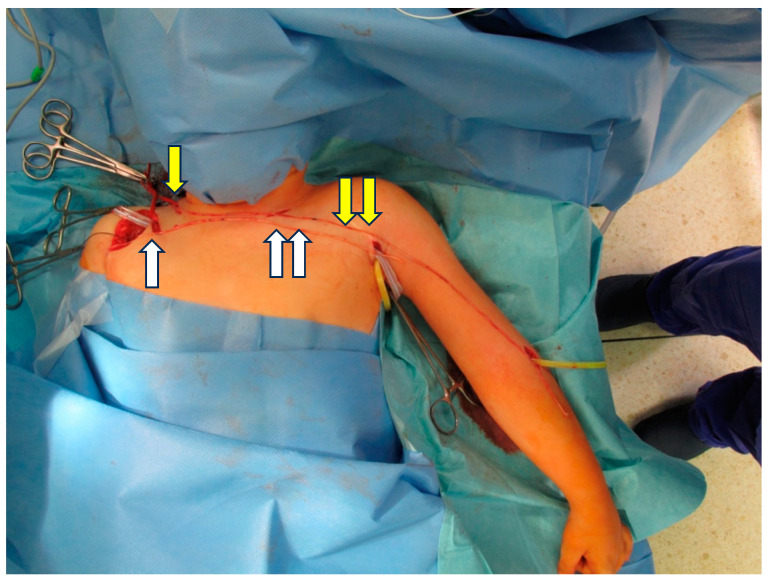
Intraoperative scenario of a contralateral transfer for reconstruction of biceps and finger flexors of the injured left arm. M. pectoralis minor motor nerve (white arrow) with a short graft (double white arrow) for biceps restoration and spinal accessory nerve (yellow arrow) with a long nerve graft (double yellow arrow) for restoration of finger flexion.

**Figure 2:**
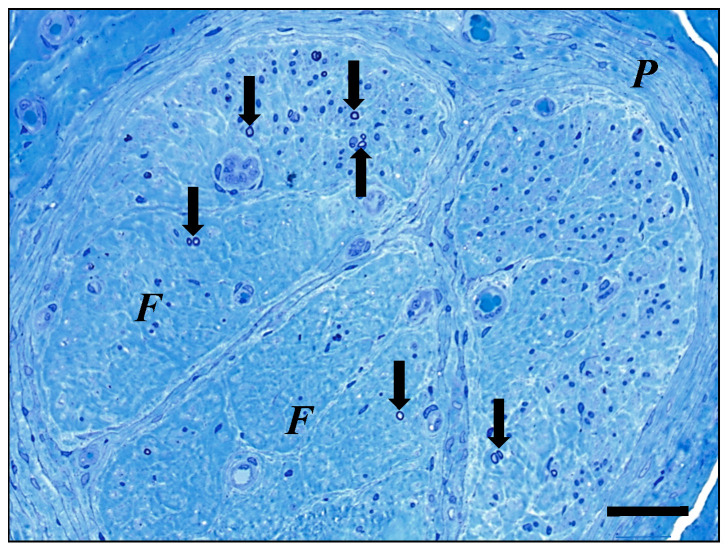
Transplanted nerve (cross-section of one nerve fascicle). Several regenerated nerve fibers (arrows) are discernable, some of which lie in small groups of two or three. The unevenly distributed fibers show small calibers and thin myelin sheaths. Note the prominent fibrosis (*F*) of large parts of the fascicle area, associated with a marked reduction of Schwann cell density. Considerable fibrotic thickening of the perineurium (*P*) can also be seen. Semi-thin section, toluidine blue stain (scale bar = 35 µm).

In the period between April 2004 and April 2023, we performed 327 neurovascular free muscle transfers (M. gracilis and M. latissimus dorsi). Twenty flaps were lost due to vascular insufficiency, and 61 muscle transfers were performed for injuries other than brachial plexus palsies. Twenty-one patients were still regarded as undergoing regenerative growth by the time of this evaluation, and seventeen patients, who mostly lived abroad, were not available for further evaluation. From the cohort of the remaining 208 patients, a direct coaptation to the donor nerve was performed in 103 cases. Additional nerve grafts were used for 105 cases. The nerves were selected according to availability since some patients had already undergone reconstructive procedures. The first-choice nerve transplant was the sural nerve, followed by the saphenous nerve. For nerve graft lengths of less than 5 cm, the muscle transfer was performed in a single stage (n = 14); however, as described above, a two-stage procedure was used for longer nerve grafts. The distal end of the nerve graft was placed into the region where it would later undergo coaptation to the motor nerve of a muscle graft during the second stage of the procedure. The distal end of the nerve was attached to the dermis with a stay suture and its location indicated for later detection by the positioning of a vessel clip (e.g., Figure 1). After the estimated regeneration time (based on a regenerating speed of 1 mm per day, plus an extra 4 weeks to account for the anticipated time required for regenerating axons to overcome the coaptation site), the distal end of the graft was biopsied as an open procedure. The biopsy was then fixed in 3.9% phosphate-buffered glutaraldehyde and processed for embedding in resin. Semi-thin transverse sections (1 µm thick) were prepared and stained with toluidine blue and paraphenylendiamine as described earlier [9]. If regenerating myelinated axons were detectable in the biopsy (or even a single axon), this was taken as confirmation that the proximal nerve coaptation was successful (e.g., Figure 2), and the muscle transfer was performed. If, however, no regenerating axons could be detected, either a failure of the nerve coaptation or a slow regeneration process was considered as a possible cause. We gave these patients a second chance and a further biopsy was scheduled to take place 3 to 6 months later. In only 3 cases was a decision made to abandon the muscle transfer procedure due to a failure to detect any regenerating axons within the distal end nerve biopsy.

Figure 1 shows the intraoperative site of a contralateral transfer, with the accessory nerve as the donor, in preparation for a biceps muscle reconstruction. The graft is transferred subcutaneously and the distal end is marked with vascular clips. 

Figure 2 shows regenerating axons present within a transverse semi-thin section that was prepared from the biopsy specimen taken from the distal end of the nerve.

The clinical evaluation for elbow flexion and extension as well as finger flexion and extension (Table 2) was performed 1.5 years post-surgery by an examiner who was independent of the surgeon in accordance with the MRC scale [10]:

M0: No contraction;
M1: Flicker or trace of contraction;
M2: Active movement with gravity eliminated;
M3: Active movement against gravity;
M4: Active movement against gravity and resistance;
M5: Normal power.

Patients were categorized according to the length of the nerve graft that was used (see Table 3).

## 3. Results

Ipsilateral donor nerve transfer was performed in 124 cases, of which the accessory nerve was used in 61 cases, the pectoralis minor nerve in 59 cases, a divided phrenic nerve in 3 cases, and the hypoglossal nerve in one case. Contralateral donor nerve transfers were used in 84 cases, of which the accessory nerve was used in 21 cases, the medial pectoral nerve in 20 cases, and the C7 motor nerve in 43 cases (see also Table 1).

Depending on the patient’s anatomy, serial grafts were required for a graft length of greater than 45 cm. This was necessary in 11 cases: 8 patients from group 4 and all 3 patients from group 5. 

Meaningful motor recovery [11] was achieved in all groups except group 5. The best motor recovery was generated by direct coaptation, with 45% of patients achieving a score of M4. When using a nerve graft, the results were marginally worse, but even following the bridging of large distances (i.e., group 4, 30–60 cm), the majority of patients (i.e., approximately 73%) achieved motor recovery levels of either M3 or M4 (Table 4). 

Of the 11 patients in whom serial grafts were used, 8 patients fell into group 4 (30–60 cm) and 3 into group 5 (60–90 cm), where a motor recovery level of M3 (group 4) was achieved in 2 cases. In the remaining nine cases, the degree of motor recovery was insufficient, being M1/M2 (Table 4).

## 4. Discussion

A majority of brachial plexus injuries are caused by road traffic accidents, mainly motorcycle accidents, resulting in polytrauma and, in a majority of cases, causing at least one root avulsion and also affecting the lower roots [12]. Such injuries with root avulsions where a contralateral nerve transfer is considered pose a particular challenge for surgical reconstruction, especially for reanimation of the hand due to the long distances that must be bridged [13,14]. This is particularly the case when contralateral nerve transfers are required in conjunction with neurovascular free functional muscle transfers, where the motor branch of the muscle graft adds extra length (10 cm in a gracilis muscle graft). This has prompted the question as to whether there is a substantial loss of axons over the distance of a long transplant or whether axonal regeneration is sufficiently robust without significant axonal loss. 

There is currently no imaging technique available that allows the intra-fascicular number of axons to be determined. A cross-sectional biopsy of a peripheral nerve will lead to complete destruction of the nerve. In the setting described here, the biopsy of the distal end of the nerve was performed to verify the success of nerve coaptation as a qualitative, rather than quantitative, examination prior to the planned muscle transfer. At present, there is only a clinical outcome that can answer the question of the regenerative potential of long nerve grafts. Our results demonstrate moderate (M3) to good (M4) regeneration in patients where long grafts have been used for nerve reconstruction. However, the prerequisite for such positive results is a vital graft bed without fibrotic scarring, as is generally the case with free tissue grafts. Even with meticulous surgical technique, some mis-direction of fascicles may occur at the coaptation site, causing a loss of regenerating axons [7,8]. Direct nerve suture provides better clinical results than transplantation, where a second coaptation site is present. This is confirmed by our data; the best clinical recovery was achieved when the motor branch of the muscle graft was directly coaptated to the donor nerve (i.e., group 1). With the use of a nerve graft (see groups 2, 3, and 4), two coaptation sites had to be overcome by regenerating axons, which still led to good clinical results. In contrast to this, only 2 out of 11 patients who received serial nerve grafts (i.e., three coaptation sites) achieved a return of motor function that reached level M3. 

The present retrospective study has been able to demonstrate that axonal regeneration is possible over long distances and can lead to clinically useful motor recovery when combined with free muscle transfers. Technically, this procedure is limited by the length of the grafts, with the use of serial nerve grafts inevitably leading to a functionally relevant loss of axons as they attempt to cross three coaptation sites. 

## Figures and Tables

**Table 1:** Donor nerves.

N. accessorius ipsilateral	61
N. accessorius contralateral	21
N. pect. med. ipsilateral	59
N. pect. med. contralateral	20
C7 contralateral	43
N. phrenicus ipsilateral	3
N. hypoglossus	1
	
	208

**Table 2:** Muscle transfers performed and the associated repair of function.

Donor Muscle	Function Repaired	Number of Cases	Male/Female	Mean Age
M. gracilis	M. biceps	104	88/16	34.6
M. gracilis	Finger flexors	63	53/10	31.7
M. gracilis	Finger extensors	31	26/5	30.2
M. gracilis	M. triceps	3	3/0	29.7
				
M. lat dorsi	M. biceps	6	5/1	35.3
M. lat dorsi	M. deltoid	1	0/1	15

**Table 3:** Patient grouping according to the length of nerve graft used.

Group	Nerve Graft	N
		
1	Direct	103
2	<5 cm	14
3	5–30 cm	20
4	30–60 cm	68
5	60–90 cm	3

**Table 4:** Post-operative clinical evaluation of free neurovascular muscle transfers (n = 276). All patients demonstrated preoperative motor function levels of M0.

Group Number (Nerve Length)	M0/M1	M2	M3	M4	M5	Sum
1 (0)	2 (1.9%)	12 (11.6%)	39 (37.8%)	47 (45.6%)	3 (2.9%)	103
2 (<5 cm)	0 (0%)	1 (7.1%)	7 (50%)	5 (35.7%)	1 (7.1%)	14
3 (5–30 cm)	2 (10%)	3 (15%)	8 (40%)	6 (30%)	1 (5%)	20
4 (30–60 cm)	7 (10.2%)	11 (16.2%)	28 (41.2%)	22 (32.3%)	0 (0%)	68
5 (60–90 cm)	2 (66%)	1 (33%)				3

## Data Availability

The original contributions presented in the study are included in the article. Further inquiries can be directed to the corresponding authors.
